# Microstructure Evolution and Mechanical Properties of 16-Layer 2195 Al-Li Alloy Components Manufactured by Additive Friction Stir Deposition

**DOI:** 10.3390/ma17235748

**Published:** 2024-11-24

**Authors:** Qinglin Liu, Ruilin Lai, Hui Wang, Yidi Li, Yunping Li, Lihua Zhan

**Affiliations:** 1 State Key Laboratory for Powder Metallurgy, Central South University, Changsha 410083, China; silin_link@csu.edu.cn (Q.L.); wanghuiii@csu.edu.cn (H.W.); 2 Research Institute of Light Alloy, Central South University, Changsha 410083, China; lairuilin@csu.edu.cn (R.L.); yjs-cast@csu.edu.cn (L.Z.); 3 State Key Laboratory of High-performance Complex Manufacturing, Central South University, Changsha 410083, China

**Keywords:** Al-Li alloy, additive friction stir deposition, heat input, microstructure evolution, thermo-mechanical behavior

## Abstract

The fabrication of multi-layer alloys by additive friction stir deposition (AFSD) results in a complicated microstructure and mechanical property evolution due to the repeated thermal inputs impacting the existing deposited layers. This work systematically studied the microstructure and mechanical properties of several areas (last layers, intermediate layers, and first layers) of a 16-layer 2195 alloy component fabricated by AFSD to ascertain the effect of repeated thermal cycling. The periodic heat input resulted in the minimal quantities of T_1_-phase only appearing in the last layers of the sample, while the θ′-phase developed a complex precipitate with the δ′ and β′ phases. The mechanical properties of the 2195 sample exhibit a gradient development related to the microstructure, with a decrease in strength and hardness from top to bottom. The samples located in the last layers show the highest microhardness of 117.0 Hv, yield strength of 296.6 MPa, ultimate tensile strength of 440.6 MPa, and elongation of 27.1%, respectively.

## 1. Introduction

Aluminum–lithium (Al-Li) alloys are an optimal selection for lightweight structural materials due to their strength, processing capabilities, and high damage tolerance [1,2,3]. The first generation was developed for weight reduction [3], through the addition of a significant quantity of lithium to traditional aerospace aluminum alloys. This resulted in a reduction in weight of approximately 6% in the engineering sector [4]. However, current metallurgical technology cannot produce alloys with the desired comprehensive performance due to impurities, particularly in terms of plasticity [5]. Second-generation Al-Li alloys have better stiffness due to advanced control of elemental composition and specific proportions. This has led to the development of several models, including 1420 and 2090. Nevertheless, the alloys still had shortcomings, including anisotropy, inadequate plasticity, and challenging processability, which hindered their application in the aerospace industry [6]. Following decades of development, the third generation (typically 2195 alloy) has achieved significantly improved anisotropy, damage tolerance, and processability of the alloys by decreasing Li content, increasing Cu content, and incorporating a variety of micro-alloying elements [3,6]. Notably, the 2195 alloy has widely been employed in the aircraft and aerospace industries as a raw material for the stiffened panel’s structure, including the fuselage and wing skins, as well as fuel bulkheads and other critical components [1,2]. As a typical third-generation Al-Li alloy, the mechanical properties of 2195 alloy are significantly influenced by the type, size, and distribution of precipitates [7,8]. To illustrate, the dominant strengthening precipitate in Al-Li alloys is the T_1_-phase (Al_2_CuLi), due to the low Li content. However, when the Li content is sufficiently high, the δ′-phase (Al_3_Li) dominates. The interactions of the T_1_ and δ′ phases with dislocations are bypassing and shearing, respectively. Consequently, the former exhibits a higher precipitate strengthening effect. Nonetheless, regulating the alloy composition and precipitated phases is frequently challenging due to the presence of the Li element with a low melting point [9,10,11].

In the traditional production of 2195 stiffened panels, the combustion loss of the li element presents a significant challenge for the casting and welding process in maintaining compositional stability [12,13,14]. The significant temperature fluctuations during the welding process can lead to the formation of substantial welding residual stress or even internal solidification cracks [15,16,17,18]. Murphy et al. [18] reported that laser welding technology tends to introduce significant residual stresses, which can result in the distortion of the component. Furthermore, the use of sophisticated equipment is necessary to ensure a close-fit subcomponent setup and to optically align the laser beam. Consequently, the operational and tooling costs are considerable, and the equipment must be utilized extensively to achieve cost-effectiveness. Friction stir welding (FSW) is a novel approach to solid connection technology in the welding of aerospace aluminum alloys. FSW is faster, uses smaller joints, and reduces the mass of aerospace structures [11,19]. Friction stir additive manufacturing (FSAM) has extended the scope of product applications. Zhao et al. [20] successfully performed friction stir additive manufacturing (FSAM) using 2 mm thick 2195 Al-Li alloy plates. A pin with three concave arc grooves and back-and-forth double-pass welding were employed to enhance material mixing throughout the nugget zone and to achieve ultimate tensile strength (UTS) of up to 348 MPa with an elongation (EL) of 9.6%. However, FSW and FSAM still have shortcomings, including insufficient joint strength, hole defects, and macroscopic gradients in mechanical properties [19,21].

Additive friction stir deposition (AFSD) is a solid-state additive manufacturing technology that effectively minimizes the issue of high-temperature burnout of Li, ensures compositional stability, and alleviates constraints on product dimensions and shape. AFSD can rapidly achieve near-net shaping of large, intricate components. In particular, for alloys with wide solidification ranges, which permit volumetric shrinkage and significant thermal contraction, AFSD can effectively prevent the formation of defects such as hot cracks, porosity, and columnar grain, which are prone to occur in traditional melting-based additive manufacturing [15,16]. AFSD induces intense rotation and extrusion of the rod or filament feed material, causing the deformation and fracture of the raw material ends. During this process, the material is heated to 50–90% of melting point [15,22,23,24] and softened for deposition under evaluated temperatures. AFSD samples, owing to the solid-state temperatures and severe plastic deformation, typically develop uniform, fine, equiaxed grains with evenly distributed precipitates, resulting in optimal density and mechanical properties [25,26,27,28]. For example, Farabi et al. [14] demonstrated the successful implementation of AFSD of Ti6Al4V alloys over a broad processing window. Changing AFSD parameters reduced the deposition temperature, shrinking β grains and refining the α phase structure. This markedly enhanced the ductility of the as-deposited material while maintaining its high strength. In this study, the Ti6Al4V alloy exhibited ductility of up to 20%, which surpassed the majority of conventional manufacturing technologies (rarely exceeding 13%), and maximum yield strength (YS) of 1010 MPa, which is among the highest observed in manufacturing technologies. Wang et al. [27] successfully repaired AA7075 plates by AFSD. The T6-treated feedstock rod showed an excellent performance level in this work; the repair specimen is filled with the mixture of the sidewall and deposited material, showing no distinct interface. The repair specimen exhibited a maximum UTS of 312.6 MPa and an EL of 9.7%. To further improve the mechanical properties of the repaired specimen, post-repair heat treatment (PRHT) was performed. After PRHT, the UTS of the repair specimen increased to 491 MPa.

Nevertheless, the majority of reported studies on AFSD technology are constrained to small-thickness samples with a limited number of layers. The introduction of AFSD technology to materials such as steels and nickel-based alloys, which are excessively hard, is challenging due to the rigidity of the deposition tool and the rapid heat accumulation [28]. Furthermore, for alloys in which the melting point of certain components is below the AFSD temperature threshold, such as AlSn20Cu, it is critical to prevent the loss of the liquid phase due to spillage and oxidation throughout deposition [29]. More importantly, the cycle thermal process of multilayer deposition, as a layer-by-layer additive manufacturing technique, significantly influences the organization and properties of the material (Especially for precipitation-strengthened Al-Li alloys). For instance, Li et al. [30] identified the layer-dependent microstructure and mechanical properties in the AFSD 2195 Al-Li sample composed of four layers. For thick components such as stiffened panel structures, the impact of repeat heat input during the AFSD process on the microstructure is significantly amplified with an increase in the number of deposited layers, hence requiring a comprehensive investigation.

In this work, a 16-layer 2195 Al-Li alloy component was prepared by AFSD with a focus on its microstructure and mechanical properties. Scanning electron microscopy (SEM), electron backscattered diffraction (EBSD), and transmission electron microscopy (TEM) were used to examine the microstructure of the layers and link the microstructure evolution to heat input history. The microhardness, tensile properties, and fracture behavior of samples from different locations of the component were analyzed to investigate the relationship with the microstructure. This research offers valuable insights and optimization avenues for additive manufacturing of multi-layer Al-Li alloy components.

## 2. Experimental Methods

The material used in this work is the commercial 2195-T34 Al-Li alloy [3,31], and its chemical composition was determined by the inductively coupled plasma optical emission spectrometer (Spectro Blue instrument ICP-OES, Kleve, Germany), as illustrated in Table 1. The raw material was cut into 250 × 50 × 7 mm³ plates as substrates and 10 × 10 × 100 mm^3^ square bars using an electrical discharge machine (DK7735, Taizhou Zhenghua CNC Machine Tool Factory, Taizhou, China). The bars were solution-treated at 520 °C for 2 h and then cooled in water.

**Figure 1:**
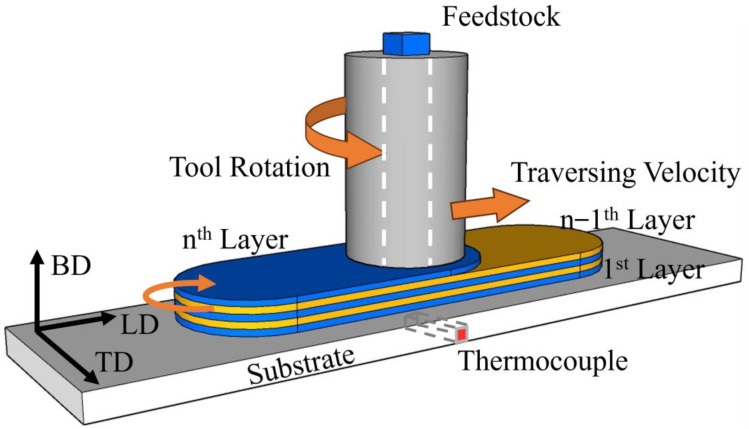
Schematic diagram of the AFSD process. The orange arrows indicate the direction of rotation and translation of the deposition tool.

AFSD experiments were conducted using the additive friction stir machine (FRC002, CSU, Changsha, China), as illustrated in Figure 1. The building direction was identified as BD, the transverse direction as TD, and the longitudinal direction as LD. A thermocouple was positioned at the center of the substrate’s lower surface to collect temperature data at a frequency of 12 points per second during the AFSD process. Before the deposition, the substrate was subjected to a preheating process of 150 °C. Each feedstock rod deposits a single layer. The tool was moved in a back-and-forth motion in the LD for densification, and water cooling was employed on the tool throughout the process. The optimal parameters of the AFSD process are as follows: tool rotation rate *ω*: 350 RPM; material feed rate *F*: 52 mm/min; traversing velocity *V*: 70 mm/min.

**Figure 2:**
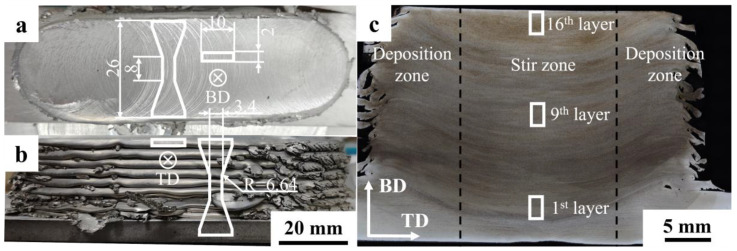
(**a**,**b**) Photographs of 16-layer 2195 alloy deposition, with the dimensions of the tensile specimens indicated in the accompanying figure; (**c**) the cross-section morphology of 2195 AFSD sample. The locations of tensile specimens, SEM, and TEM characterization are shown as solid lines.

The microhardness mapping of the cross-section specimen parallel to the TD-BD plane was quantified using a hardness tester (Huayin instrument 200HV-5, Laizhou Huayin Test Instrument Company, Laizhou, China) at a load of 2.5 kg and an indentation spacing of 1.5 × 1.5 mm². Tensile properties in the BD and TD were evaluated using a tensile tester (Instron instrument 5969 electronic universal material tester, Instron, MA, USA) with a tensile rate of 0.5 mm/min. The sampling positions of the tensile specimens in the BD and TD are shown in Figure 2a,b.

An optical microscope (BX53M, Olympus, Shinjuku City, Japan) was employed to characterize the AFSD sample cross-section morphology. The samples for metallographic observation were mechanically polished using an oxide polishing suspension (OPS) and subsequently etched with Keller’s reagent for 20 s. The distribution of precipitates of the samples was obtained using an SEM (FEI instrument Quanta 650, Thermo Fisher Scientific Inc, MA, USA) equipped with an EBSD detector (Oxford instrument, Oxford, UK). The type and morphologies of the precipitates were characterized using a TEM (FEI instrument Talos F200 S, Thermo Fisher Scientific Inc, MA, USA) with an energy dispersive spectrometer (EDS). The samples intended for TEM characterization were electrolytically polished using a twin-jet electro-polisher (Kamaray instrument DJ2000, Dedo, Beijing, China), with a ratio of 30% HNO_3_: 70% CH_3_OH mixture. Polishing parameters are −25 °C and 12 V.

## 3. Results

### 3.1. Thermal History

**Figure 3:**
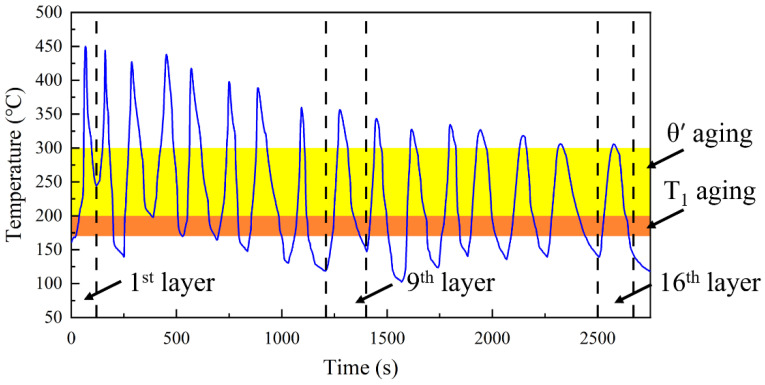
The curve of temperature versus time at the center of the substrate. The deposited layers for microstructural characterization are indicated by dashed lines; the aging temperatures are highlighted in orange and yellow.

During the AFSD process, the temperature in the center region exhibits periodic variations with time, as illustrated in Figure 3. The temperature curve of the deposition center for the initial two temperature cycles is markedly sharp and exhibits a high peaking temperature of 450 °C. As the number of deposited layers increases, the peaking temperature decreases in each cycle. The temperature ranges commonly employed in industrial engineering for the T_1_ and θ′ phases are indicated on the graph by orange and yellow markers, respectively [30,32,33,34]. It is noteworthy that the effective nucleation temperatures of the primary reinforcing phases of 2195 (T_1_ and θ′ phases) are present for only 15 s in the ramp-up section of each cycle [31,34,35,36]. Subsequently, the dissolution temperature interval and the effective nucleation temperature in the cool-down section are reached, which, once again, occur for only 20 s.

### 3.2. Gradient Microstructure

Figure 2 shows images of the 2195 Al-Li alloy sample prepared via AFSD. A total of 16 layers of the sample were produced with an average single layer size of approximately 120 × 38 × 1.4 mm³ (Figure 2a,b). Consequently, the flashes in the mid-section of the samples were lapped with downward flow and exhibited cracking at the ends due to material overfilling. The morphology of the cross-section of the sample along the BD-TD plane in Figure 2c shows that the width of the effective bonding zone is 34–35 mm, with the individual deposited layers decreasing from the bottom to the top. The AFSD sample can be divided into three zones based on the stirring range of the deposition tool protrusions: the central stir zone and the deposition zones flanking each side [37]. The stirring action of the protrusions and the feedstock penetrated approximately 4 mm below the surface of the deposited layer, creating a concave of 4 mm in the center zone of each deposited layer. Additionally, the interlayer interface of the stirring zone is broken by the stirring action of the protrusions, forming a jagged interface. The materials of the adjacent 2–3 layers were fully mixed under the cross-layer stirring action of the protrusions, thereby avoiding the fracture-sensitive zone caused by the interface.

**Figure 4:**
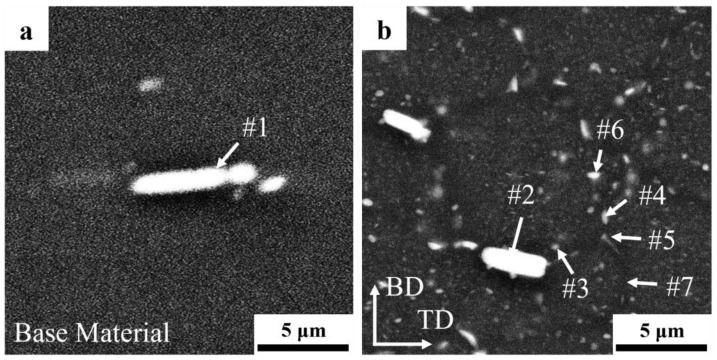
Precipitates of (**a**) feedstock and (**b**) the AFSD sample. The locations of EDS characterization are shown as arrows and numbers.

The precipitates of the feedstock and AFSD samples were characterized by EDS, and the results are presented in Table 2. After solid solution treatment, a minimal quantity of insoluble rich-Cu/Fe phases [31,35] still existed in the matrix (Figure 4a). These included needle-like and ellipsoidal silver-rich phases, as well as ellipsoidal Al-Cu phases [30,31,35], as illustrated in Figure 4b. The scanning of the coarsened precipitates within the crystal revealed that they are primarily ellipsoidal Al-Cu phases with Al-Cu-Zr phases. It is noteworthy that the rich-Cu/Fe phase in the deposited material is smaller in size and more inhomogeneous in shape, which suggests that it was fragmented during the AFSD process. Additionally, hemispherical precipitates are observed to be growing in proximity to the boundary of the rich-Cu/Fe phase.

**Figure 5:**
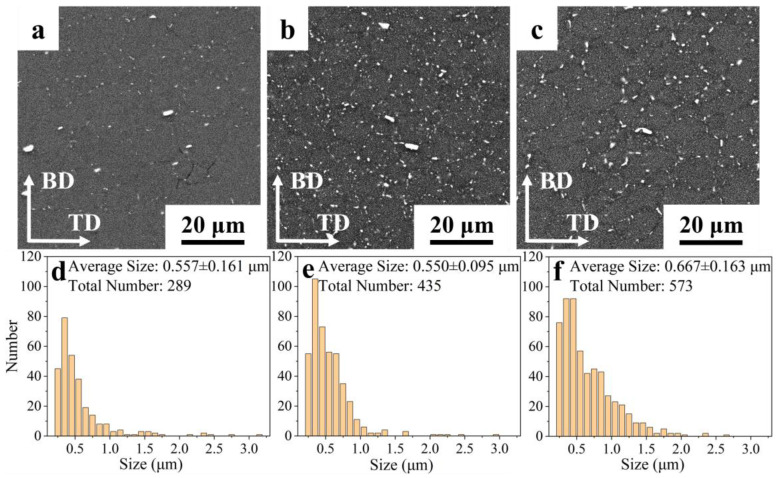
(**a**–**c**) SEM images under BSE mode and (**d**–**f**) corresponding grain size distribution, and the statistical number of precipitates in the (**a**,**d**) 16th layer, (**b**,**e**) 9th layer, and (**c**,**f**) 1st layer.

The statistical results for the size and distribution of the precipitates were obtained from the center region of the top (16th layer), the middle (9th layer), and the bottom (1st layer) of the sample (Figure 5). Figure 5a illustrates the central region of the 16th layer, wherein the freshly deposited material was preserved due to the absence of subsequent thermal cycling. It can be observed that some large, irregularly shaped rich-Cu/Fe phases are primarily distributed at the grain boundaries (GBs), along with a modest quantity of smaller precipitates in the form of rods and ellipsoids. Following the application of eight subsequent periodic heat inputs, the small-sized precipitates at the GBs exhibited a rapid increase and coarsening. Additionally, some of the precipitates were observed to grow attached to the boundaries of the large-sized rich-Cu/Fe phases. A slight coarsening of the Al-Cu phase was observed within the grain, as illustrated in Figure 5b. In the initially deposited layer, after 15 subsequent periodic heat inputs, both the GBs and the intercrystalline precipitates exhibited notable coarsening, while the density of the intercrystalline precipitates appeared to increase further, as illustrated in Figure 5c,f.

**Figure 6:**
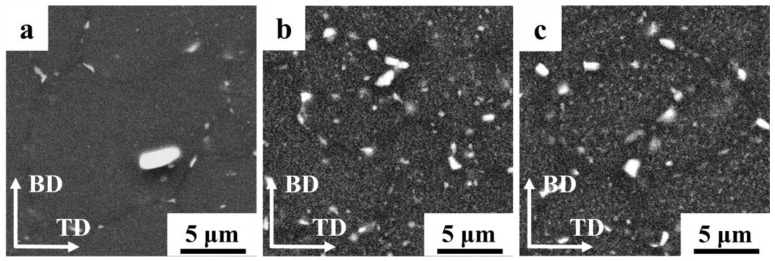
High-magnification SEM images of (**a**) 16th layer, (**b**) 9th layer, and (**c**) 1st layer.

Figure 6 illustrates the SEM morphology of grains of the 16th, 9th, and 1st layers, showing visible GBs visible under BSE mode. The rich-Cu/Fe phases tend to localize at GBs. In the 16th layer, coarsened rod-like precipitates were identified at the GBs. In the ninth layer, the presence of several distinct precipitates was observed near the GBs. Moreover, the precipitation and coarsening behaviors of the ninth and first layers exhibited a notable progression with the increase in the number of thermal cycles, which aligns with the reduction in the solid solution Cu in Table 3. Although the results of the EDS analysis are limited by the accuracy of the detector, the EDS results of different deposited layers are sufficient to demonstrate, at the qualitative level, that the Cu content of the 16th layer is essentially consistent with the average Cu content presented in Table 2. Conversely, the Cu content dissolved in the matrix of the 9th layer exhibits a notable decline and is close to that in the 1st layer. The increase in Cu-rich precipitates and decrease in solid solution Cu content may be related to the increase in the number of thermal cycles.

**Figure 7:**
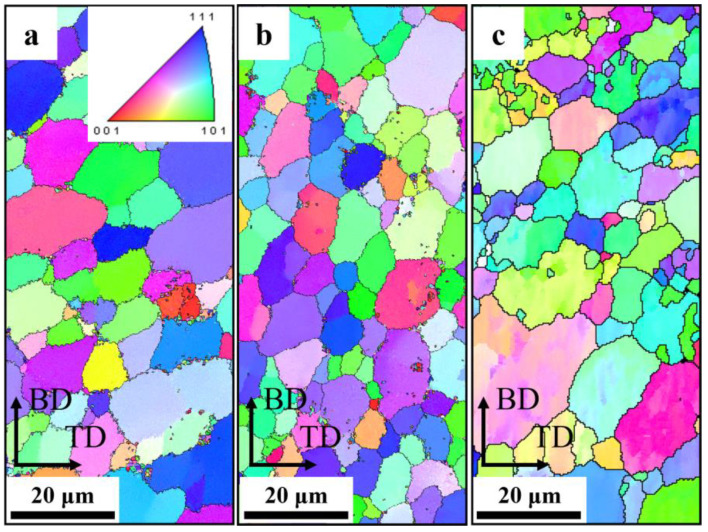
The IPF maps of (**a**) the 16th layer, (**b**) 9th layer, and (**c**) 1st layer.

**Figure 8:**
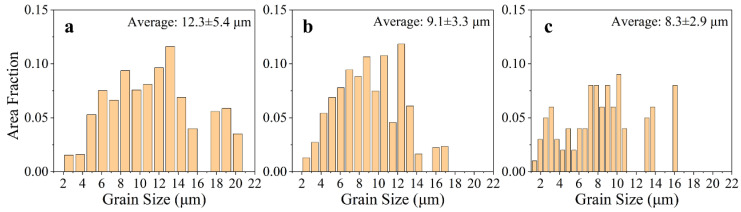
Grain size distribution in the (**a**) 16th layer, (**b**) 9th layer, and (**c**) 1st layer. The average grain sizes are labeled in the upper right.

Based on the evolution of solid solution elements and microstructures of different deposited layers, the central region of the 16th, 9th, and 1st layers was selected for EBSD characterization. The AFSD samples consisted of uniformly oriented equiaxial crystals, and the long axis of the grains exhibited a slight deflection to 45°, which demonstrated the typical features of the AFSD process (Figure 7) [38]. As a consequence of the vertical stirring action of the deposition tool, the depressed region in the center of 1st layer penetrates deeply into the substrate, resulting in the mixing of the substrate material with the deposited material. As a consequence of the lower thermo-mechanical action exerted on the substrate grains in comparison to the deposited layers, the dynamic recrystallization process is insufficient, resulting in larger sizes and intra-granular orientation differences, as illustrated in the lower part of Figure 7c. Figure 8 depicts the grain size and distribution statistics in the center region of the three deposited layers. To exclude the interference of large-sized grains of the substrate, the statistics of 1st layer were filtered. The average grain size and distribution range of the AFSD samples exhibited a gradient change from the bottom upwards. However, the difference in grain size between the 16th layer and the 1st layer was minimal and insufficient to significantly affect the mechanical properties of the samples [39,40].

**Figure 9:**
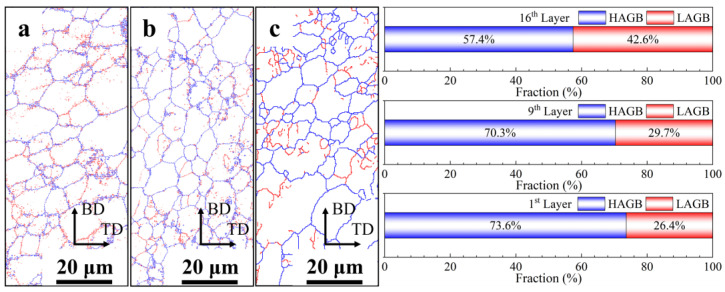
The distribution of GBs (with high-angle grain boundaries (HAGBs) represented by blue lines and LAGBs represented by red lines) and percentage of them in (**a**) 16th layer, (**b**) 9th layer, and (**c**) 1st layer.

To gain further insight into the evolution of grain size, the GB types of each deposited layer were characterized, as illustrated in Figure 9. At the 16th layer, while the individual grains are oriented in the same direction, a high concentration of low-angle grain boundaries (LAGBs) is observed within the grains, representing the highest percentage at 42.6%. This suggests that high energy levels persist in this region. As the number of thermal cycles increases, the prevalence of LAGBs is markedly diminished at the 9th layer, with the majority confined to the grains surrounding the disrupted rich-Cu/Fe phase. It is noteworthy that the proportion and distribution of LAGBs in the 9th layer and 1st layer are similar, which means the LAGBs do not decrease further with the increase in the number of thermal cycles. This suggests that the deposited layer has essentially exhausted its energy and entered a steady stage after the 8th–9th thermal cycles.

**Figure 10:**
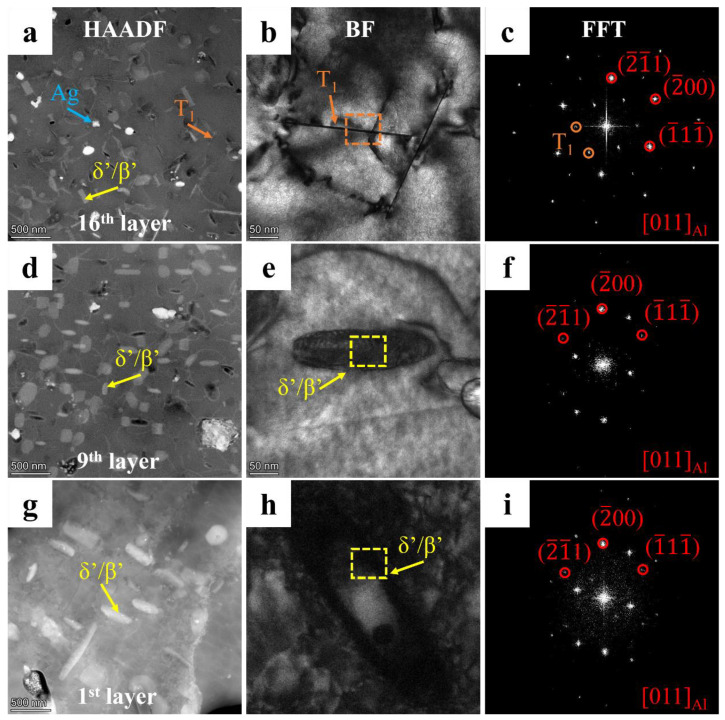
(**a**,**d**,**g**) HAADF images, (**b**,**e**,**h**) BF images, and (**c**,**f**,**i**) corresponding (insert) FFT pattern of (**a**–**c**) 16th layer, (**d**–**f**) 9th layer, and (**g**–**i**) 1st layer samples taken along [011] Al zone axis.

**Figure 11:**
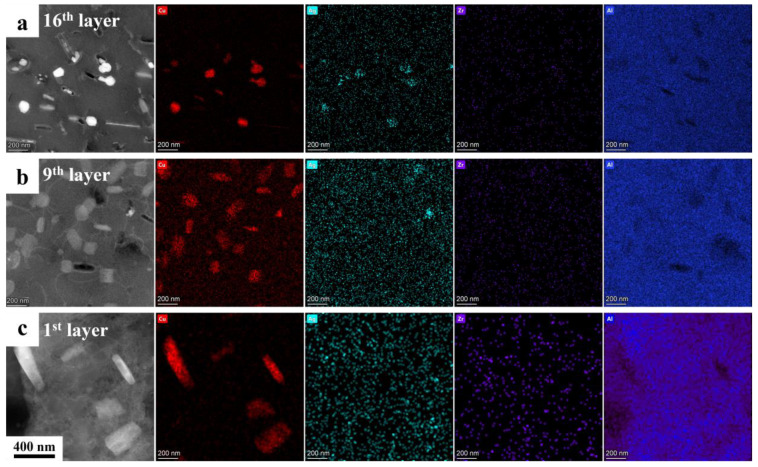
HAADF images and corresponding EDX of (**a**) 16th layer, (**b**) 9th layer, and (**c**) 1st layer samples taken along [011] Al zone axis.

To gain further insight into the influence of microstructure evolution on the strengthening mechanism, it is essential to conduct a detailed TEM characterization of the AFSD samples. Figure 10 depicts the HAADF images of the center region of layers 16, 9, and 1 along the [110] regional axis, the corresponding bright field images, and the fast Fourier transform patterns. In the 16th layer, a considerable number of clostridial precipitates and a somewhat smaller number of needle-like precipitates, in addition to clustered spherical particles, are observed. The FFT mode facilitated the identification of two distinct precipitate types: the T_1_ phase (orientation relation: (0001) T_1_//(111) Al, [1010] T_1_//[110] Al), and the δ′/β′ phase (Al_3_(Li, Zr) [30,41]). Of particular note is the T_1_ phase, which exhibits an elongated and needle-like morphology, displaying a parallel alignment along two distinct axes with an angle of 109.5°. This phase is highlighted by orange arrows in Figure 10b. Furthermore, the δ′/β′ phases are extensively distributed throughout all deposited layers. However, an increase in fragmentation is observed in the 16th layer, as indicated by the yellow arrows. In the 9th layer, the T_1_ phase, which underwent eight additional thermal cycles and exhibited a lower dissolution temperature, was no longer present. Only the δ′/β′ phase, which had increased significantly in size and number, remained, and the fragmentation ratio was markedly lower. It can be inferred that the fragmentation of the δ′/β′ phases is a consequence of the violent stirring that occurs during the AFSD process. In addition, it is also evident that these phases are capable of healing during subsequent thermal cycles. In the first layer, the size of the δ′/β′ phases continues to increase, but the quantity is reduced, and a distinct shell structure emerges. This transformation could be related to the unique thermal cycling of AFSD. The X-ray energy spectroscopy (EDX) analysis of the three deposited layers reveals that the predominant strengthening element, copper, is enriched in the precipitates, which corresponds to the results of the scans of the matrix of each layer (Figure 11). The microalloying element Ag is homogenized by thermal cycling, and the clustered spherical particles observed in the 16th layer are no longer present in the underlying layers (Figure 10a and Figure 11).

### 3.3. Mechanical Properties

**Figure 12:**
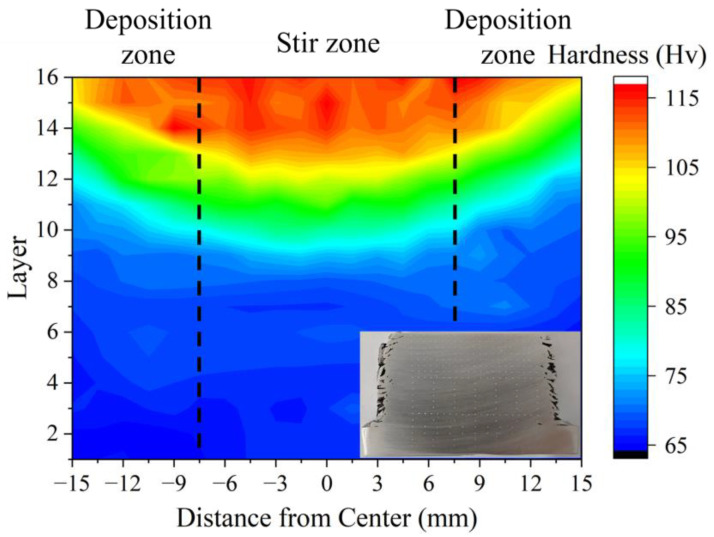
Hardness distribution mapping in the cross-section of the AFSD sample; the actual map of measurements is attached at the bottom right corner. The AFSD sample is in the first layers in layers 1–9, where the microhardness is homogeneous. Consequently, the sampling locations in this region are increased accordingly.

Figure 12 illustrates the microhardness mapping of the complete cross-section of the AFSD sample. The hardness distribution, in a certain manner, indicates the evolution in the microstructure and properties of the layers resulting from the cycle thermal-input process of the AFSD cycle [42,43]. In particular, the 16th layer exhibits the highest hardness of 117.0 HV, while the first layer demonstrates the lowest hardness of 65.3 HV. Notably, the latter is below the microhardness of the base material, which is 133.6 HV. It was observed that the microhardness does not vary linearly with the number of thermal cycles. Rather, it was found to maintain a high hardness at the top in layers 14–16. From the 13th layer onwards, there was a noticeable decay in the microhardness, which exhibited a uniform low-hardness steady state in layers 1–9. The sample can be divided into three intervals from top to bottom, depending on the hardness level, which are 105–117 Hv, 80–104 Hv, and 65–79 Hv, respectively. The boundaries of each interval exhibit a concavity that matches the interface of the deposited layers. This indicates that the microhardness is at the same level inside the individual deposited layers.

**Figure 13:**
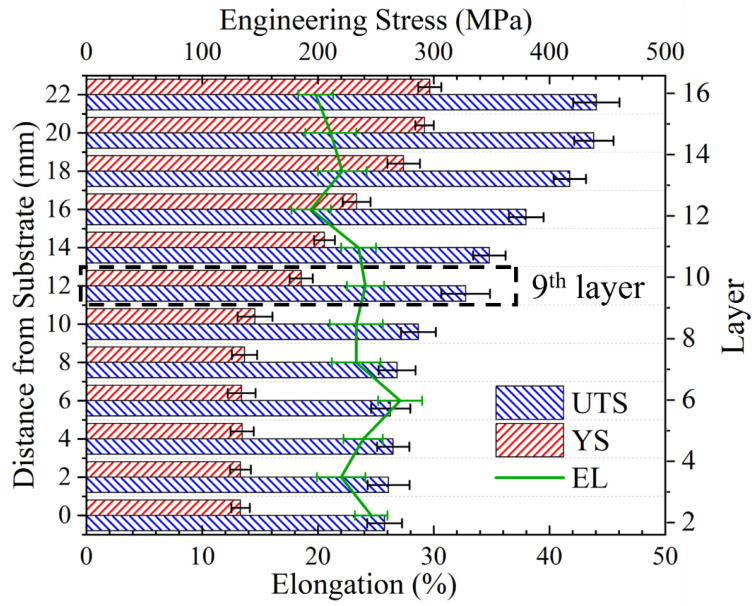
Distribution of tensile properties in different layers; the substrate surface is aligned with 2nd layer due to the depression. The tensile specimen in the 9th layer is used to compare the anisotropy.

The tensile properties of the various deposited layers along the TD were compared, as illustrated in Figure 13. The results demonstrated a comparable trend to that observed in the microhardness. The UTS and YS are optimal at the top, with values of 440.6 MPa and 296.6 MPa, respectively. These values remain stable within 4 mm of the top, i.e., in layers 14–16. In contrast, at the location of layers 13–14, the UTS decreases linearly from 438.3 MPa to 268.3 MPa in layers 7–8. After this point, the strength remains stable in layers 1–7. The YS exhibits a similar trend to that of the UTS, demonstrating a stabilization at 296.6 MPa in layers 14–16 and a subsequent decrease from 274.0 MPa in the 13th layer to 136.5 MPa in layers 7–8, after which it remains stable. The EL of each deposited layer remained within an optimal range of 19.4% to 27.1%. The lowest EL was observed at the 16th layer, at 19.6%. After that, a linear increase was noted, reaching 23.5% in the 10th layer. The subsequent layers exhibited a stable EL in layers 1–10.

**Figure 14:**
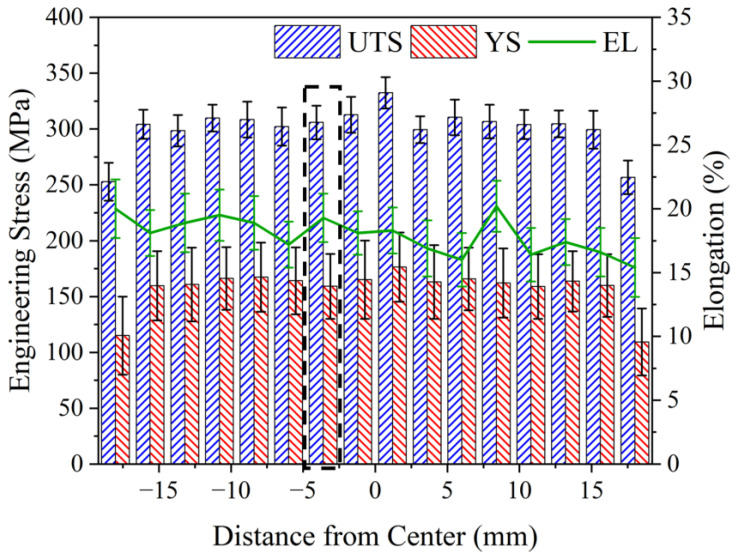
Distribution of tensile properties in BD of AFSD build. The tensile specimen in the stir zone is used to compare the anisotropy.

Figure 14 presents a comparative analysis of the tensile properties along the BD between the stir zone in the center and the deposition zones on both sides. The tensile properties remained stable in the bonding zone, exhibiting a decrease only at the defects at the edges. The UTS and YS are superior in the stir zone, with the maximum values of 332.4 MPa and 176.3 MPa, respectively. In the deposition zone on both sides, these values are stabilized at 299.4–309.9 MPa and 159–167.3 MPa, respectively. EL is stabilized in the range of 16–20.2% throughout the bonding zone. Furthermore, no significant difference is observed between the stir zone and the deposition zones on both sides.

**Figure 15:**
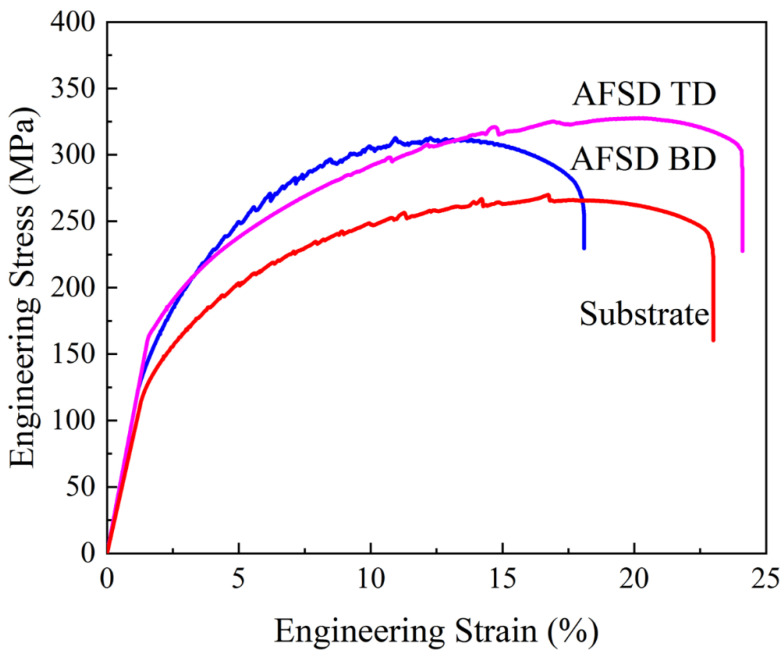
Tensile curves of TD and BD specimens in the stir zone of the 9th layer (depicted as a box in Figure 13 and Figure 14) and substrate after the process.

A comparison of the tensile curves of 9th layer along the TD and BD and the curve of the substrate along the TD after AFSD process reveals that the UTS of the deposited material in the TD and BD is relatively close, at 327.6 MPa and 312.8 MPa, respectively (Figure 15). The YS of ninth layer along the TD is slightly superior to that in the BD, at 185.6 MPa and 165.1 MPa, respectively. Additionally, the advantage in the TD is more pronounced in terms of EL, at 24.1% and 18.1%, respectively. The UTS and YS of the substrates were found to be inferior to those of the AFSD samples, which exhibited values of 269.9 MPa and 146.8 MPa, respectively.

**Figure 16:**
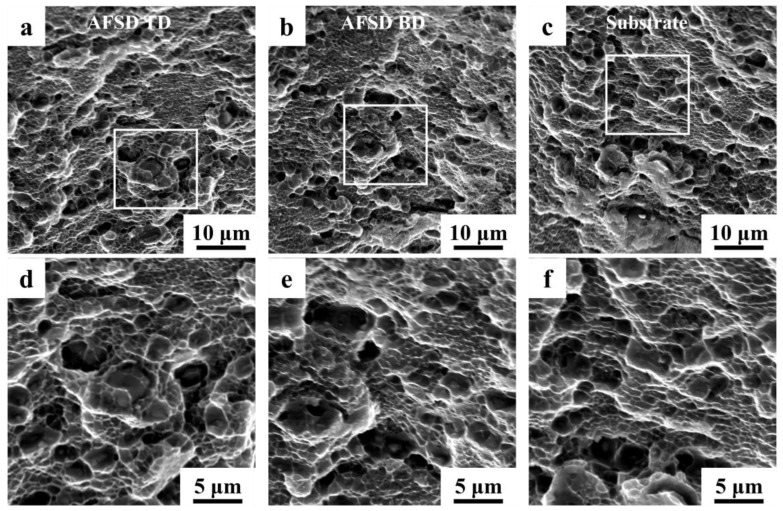
The fractography of tensile specimens in the (**a**,**d**) TD and (**b**,**e**) BD in the stir zone of the 9th layer and the (**c**,**f**) substrate.

A series of tensile specimens were subjected to analysis to ascertain the characteristics of tensile fractures in different directions, as illustrated in Figure 16. The tensile fracture of the ninth layer along the TD and BD exhibited minimal discrepancy, both manifesting as complete ductile fractures. The fractures in both directions exhibit dimples of comparable dimensions, and precipitates are discernible at the bottom (Figure 16d,e). Moreover, the fractures are uniform and continuous, rendering the interface between adjacent deposited layers challenging to discern. The fracture of the substrate along the TD exhibits a distinct morphology, characterized by smaller dimples and a structure analogous to the cleavage plane.

## 4. Discussion

### 4.1. Microstructure and Mechanical Property Evolution Mechanisms During Each Thermal Cycle

In contrast to conventional heat treatment processes, each thermal cycle during AFSD is a rapid, incomplete dynamic heat treatment. The observed effects on grain size and morphology can be summarized as a combination of simultaneous and insufficient dynamic recovery, coupled with continuous dynamic recrystallization. Previous research [14] revealed that, due to the limited duration of a single thermal cycle, although dislocations within the grains were aggregated to form LAGBs, the majority of these LAGBs were challenging to transform into HAGBs. This results in notable grain size refinement and reduced intragranular orientation differences (Figure 7). However, the deposited layer that has undergone 1–3 thermal cycles still exhibits considerable residual energy (Figure 9). Furthermore, the refinement of grains diminishes the texture characteristics of the base material, resulting in a reduction in the texture intensity of the deposited layer [14,44].

In addition, the effect of the thermal cycle on the precipitation behavior of the alloy is more intricate. The elevated peaking temperature within a single thermal cycle (Figure 3), which surpasses the dissolution temperature of the principal precipitates [2,9], results in the dissolution of the majority of enhanced phases during the heating stage, particularly the T_1_ phase, which has a lower dissolution temperature [23,30]. Nevertheless, the limited duration of each thermal cycle resulted in larger precipitates with higher dissolution temperatures being partially retained until the temperatures dropped to the effective precipitation range [23,35]. Furthermore, the preferential precipitation of high dissolution temperature precipitates results in a large proportion of solid solution elements being depleted. This hinders the nucleation and growth of subsequent low-dissolution temperature precipitates [36]. Consequently, this competition mechanism gives rise to the tendency for precipitates to coarsen rather than increase in number during thermal cycling [14,23].

### 4.2. Microstructure and Mechanical Property Evolution During Thermo-Mechanical Based AFSD Process

The heat production during AFSD is dependent upon the combination of the feed rate, tool rotational speed, and tool traversing velocity [44]. During the AFSD process, the grain sizes of the dynamic recrystallization are primarily influenced by the thermal input of the deposition tool [45,46]. The deposition tool undergoes the AFSD process 16 times for the top last layers, resulting in higher temperatures and a larger size of dynamic recrystallization. Furthermore, the trailing edge of the tool introduces an additional plastic deformation to the deposited layer, resulting in the formation of a distinctive onion ring structure [46]. This gives rise to an inhomogeneous recrystallization behavior in the top grains, which exhibit a broader grain size distribution (Figure 8). Notably, the grain sizes of the deposited layers are significantly smaller than those of the feedstock and substrate, which results in an appreciable fine-grain strengthening mechanism [15,37,46]. Given the high number of deposited layers in this work, the deposition tool exerts influence not only on the layer currently in the deposition but also on the already deposited layers, imposing a complex thermo-mechanical behavior. In instances where the height of the protrusions exceeds the thickness of the deposited layers, material flow through multiple deposited layers occurs in the region situated beneath the deposition center [20,37]. Consequently, precipitation–dissolution cycling and dynamic recrystallization persist in these deposited layers, particularly in the stir zone. The thermal input-induced microstructure evolution is continuously reset, and the microstructure and mechanical properties can be maintained in the fresh deposition completion state. According to the temperature curve, the deposition centers could reach 500 °C [22], which is close to the solid solution temperature of 2195. The high-temperature cycling and material flow result in Cu being more fully dissolved in the Al matrix, thereby providing a larger solid-solution strengthening mechanism. Furthermore, a minor quantity of T_1_ phase is produced in this stage, which is the principal strengthening phase of 2195 and can provide a substantial precipitation strengthening mechanism. As a result of the multiple strengthening mechanisms, layers in the last layers exhibit optimal hardness and YS while maintaining good ductility. In this work, the protrusion of the deposition tool can affect approximately 4 mm below the surface. Therefore, layers 14–16 can be defined as the last layers after the AFSD process.

The area below the last layers is subjected to a greater number of thermal cycles. Due to the sufficient distance to the protrusions, this area is no longer subjected to the longitudinal material flow and begins to evolve its microstructure and mechanical properties under the influence of periodic heat input until the residual energy is depleted. As a result of the reduction in peak temperature and the absence of fresh material from the last layers, a significant quantity of δ′/β′ phases can nucleate effectively and expand in the subsequent thermal cycles. The enrichment of Cu in the composited phases allows us to infer that δ′ not only combines with β′ but also forms a coarse ternary composite structure with θ′ [35,41,47]. Precipitates with higher dissolution temperatures and larger sizes are more likely to survive the heating process. Consequently, the precipitates tend to coarsen rather than increase in number during thermal cycling. It has been demonstrated that multiple rapid periodic thermal inputs lead to the coarsening of the precipitate and a large depletion of the reinforcing elements that are dissolved in the matrix [47,48]. The T_1_ phase exhibits a lower nucleation temperature, and at higher temperatures, Cu is enriched in the more stable θ′ phase, leading to the dissolution of the T_1_ phase in the matrix during subsequent thermal cycles. The grains in this region undergo rapid migration and merging of low-angle GBs under the influence of thermal cycling, resulting in further homogenization of the grain size. However, following the intense dynamic recrystallization process, the residual energy within the grains is insufficient for the subsequent static recrystallization [45,46]. This part of the region can be defined as the intermediate layers. In the present work, this corresponds to layers 8–13, where a continued decrease in microhardness and tensile properties in the TD is observed.

In the lowermost layers of deposition, following a sufficient number of thermal cycles, the dislocations and low-angle GBs are largely depleted, and the majority of the solid-solution elements in the matrix are enriched in the coarsened precipitates. This results in the stabilization of mechanical properties at a lower level and the minimization of differences in the GBs type, grain size, distribution, and mechanical properties of the various layers of deposition. Consequently, the microstructure of the 9th layer and 1st layer exhibits minimal disparity. The observed decrease in hardness and tensile strength of the bottom deposited layers can be attributed to the weakening of the microstructure due to periodic heat input. As the deposition center moves away, the peaking temperature of the thermal cycle gradually decreases to 300 °C, and the dissolution of the precipitate and the migration of solid solution elements are suppressed [35,41]. Consequently, both the solid-solution strengthening mechanism and the precipitation strengthening mechanism are significantly compromised, which in turn affects dislocation motion [7,36,49]. This part of the region can be defined as the first layers. In this work, this corresponds to layers 1–7.

### 4.3. Anisotropy Mechanical Properties of AFSD Sample

The process characteristic of the AFSD layer-by-layer deposition inevitably results in the introduction of interlayer interfacial defects to the sample. These include oxidation upon contact with air, which occurs at a periodic distribution in the BD at 1.4 mm intervals in this work. Furthermore, due to the pronounced temperature sensitivity of the plastic deformation of 2195, a torsional weaving gradient analogous to that observed in FSW is also formed along the BD as the heat of the deposition tool accumulates [30,40,46]. Accordingly, for AFSD specimens within the same region, the mechanical properties, particularly the tensile properties, when evaluated along disparate directions, will demonstrate anisotropy as a consequence of the discrepancy in defect distribution. As illustrated in Figure 15, despite the protrusions facilitating substantial interfacial fragmentation and material flow, the UTS, YS, and EL along the BD of the ninth layer remain 5%, 11%, and 25% lower, respectively, in comparison to the properties observed along the TD. It can be demonstrated that the introduction of protrusions with dimensions larger than the thickness of the deposition layer into the deposition tool successfully diminishes the anisotropy between BD and TD. The samples display a homogeneous microstructure, particularly in the stir zone, resulting from the crushing effect of the protrusions and the promotion of material flow and mixing in multiple directions [46]. Joint line remnant (JLR) and interface voids, commonly found in FSAM and AFSD with flat-end deposition tools [20,50], are not observed in this work (Figure 16). Moreover, the tensile properties of the AFSD samples exhibit an approximately 20% enhancement in comparison to those of the substrates. This superiority derives from the fact that the substrate is subjected to the same thermal history as the initial deposited layer, but is not affected by the grain refinement induced by the deposition process [30,33,37]. Consequently, the substrate retains the original coarse grains, while the distribution of precipitates and solid solution elements is identical to that of the initial layer. The coarse grains and precipitates, along with a lower dissolution of Cu elements in the matrix, are the key factors leading to the degradation of the substrate properties.

## 5. Conclusions

In this study, a large-scaled 2195 Al-Li alloy component with 16 layers was prepared by the AFSD process. The influence of periodic heat input during AFSD on the development of microstructure and mechanical properties was examined. The main conclusions are as follows:

1. The thermo-mechanical behavior during the AFSD affects the evolution of microstructure and mechanical properties of the 2195 Al-Li alloy which can be classified into three areas. The last layers experiences stirring action and 2–3 thermal cycles. The intermediate layers experiences 4–9 thermal cycles. The first layers experiences 10–16 thermal cycles.
2. The material flow through the layers can initiate a reset of the microstructure evolution caused by periodic heat input. The influence of periodic heat input results in the homogenization of grain size, the coarsening of the δ′/β′/θ′ ternary composite precipitate, and the dissolution of the T_1_ phase.
3. The samples located in the last layers show superior mechanical properties compared to those of the intermediate layers and the first layers. Specifically, the samples located in the last layers show the highest microhardness of 117.0 Hv, YS of 296.6 MPa, UTS of 440.6 MPa, and EL of 27.1%, respectively.
4. Despite the broken interlayer interface, the tensile properties in the BD were found to be diminished. The broken interface was aligned with the TD-LD plane, so the tensile properties in the TD remained unaffected. The tensile properties in the BD were higher in the stir zone, due to the interface in the deposition zone not being broken.

## Figures and Tables

**Table 1:** Chemical composition of the 2195 alloy used in this study (wt.%).

Elements	Cu	Li	Mg	Ag	Zr	Fe	Ti	Al
wt.%	3.89	0.96	0.40	0.35	0.13	0.045	0.037	Bal.

**Table 2:** EDS results (at. %) and possible precipitates correspond to points marked in Figure 4.

Locations of EDS	Al	Cu	Fe	Mg	Ag	Zr	Precipitates
#1	81.1	14.1	4.8				τ_2_
#2	78.7	15.4	5.6	0.4			τ_2_
#3	93.6	5.8		0.6			θ′/S′
#4	91.6	6.7		1.2	0.4		T_2_
#5	93.1	4.3		2.0	0.6		T_2_
#6	90.9	7.8		0.6		0.2	δ′/β′/θ′
#7	98.2	1.2		0.6			Matrix

**Table 3:** The percentage of Cu in the matrix of the 16th, 9th, and 1st layers.

Layer	16	9	1
at. %	1.7	1.2	1.2

## Data Availability

The data presented in this study are available on request from the corresponding author.
